# A prediction model based on digital breast pathology image information

**DOI:** 10.1371/journal.pone.0294923

**Published:** 2024-05-17

**Authors:** Guoxin Sun, Liying Cai, Xiong Yan, Weihong Nie, Xin Liu, Jing Xu, Xiao Zou

**Affiliations:** 1 School of Clinical Medicine, Qingdao University, Qingdao, China; 2 College of Nursing and Rehabilitation, North China University of Science and Technology, Tangshan City, China; 3 Department of Pathology, Qingdao Central Hospital, Qingdao, China; 4 Department of Breast Surgery, Xiangdong Hospital Affiliated to Hunan Normal University, Hunan, China; University of Pisa, ITALY

## Abstract

**Background:**

The workload of breast cancer pathological diagnosis is very heavy. The purpose of this study is to establish a nomogram model based on pathological images to predict the benign and malignant nature of breast diseases and to validate its predictive performance.

**Methods:**

In retrospect, a total of 2,723 H&E-stained pathological images were collected from 1,474 patients at Qingdao Central Hospital between 2019 and 2022. The dataset consisted of 509 benign tumor images (adenosis and fibroadenoma) and 2,214 malignant tumor images (infiltrating ductal carcinoma). The images were divided into a training set (1,907) and a validation set (816). Python3.7 was used to extract the values of the R channel, G channel, B channel, and one-dimensional information entropy from all images. Multivariable logistic regression was used to select variables and establish the breast tissue pathological image prediction model.

**Results:**

The R channel value, B channel value, and one-dimensional information entropy of the images were identified as independent predictive factors for the classification of benign and malignant pathological images (P < 0.05). The area under the curve (AUC) of the nomogram model in the training set was 0.889 (95% CI: 0.869, 0.909), and the AUC in the validation set was 0.838 (95% CI: 0.7980.877). The calibration curve results showed that the calibration curve of this nomogram model was close to the ideal curve. The decision curve results indicated that the predictive model curve had a high value for auxiliary diagnosis.

**Conclusion:**

The nomogram model for the prediction of benign and malignant breast diseases based on pathological images demonstrates good predictive performance. This model can assist in the diagnosis of breast tissue pathological images.

## Introduction

According to statistical data, the number of new cases of breast cancer worldwide was approximately 2.2 million in 2020, making it the most common cancer globally [1]. Pathology is the gold standard for diagnosis, but there is a shortage of pathologists, and their levels of expertise vary widely. Even among experienced pathologists, there is significant subjective variability. Although research on artificial intelligence diagnosis is now widely conducted [2], the accuracy of existing artificial intelligence pathology diagnosis products cannot meet the diagnostic needs of clinical practice due to the high accuracy requirements of pathology diagnosis. Currently, there are no artificial intelligence pathology diagnostic products suitable for clinical applications [3]. In response to this situation, we suggest that pathologists should use predictive models to analyze the pathological features of tumors before making a diagnosis. Subsequently, tumor tissue slides can be handed over to advanced pathologists for diagnosis, while benign conditions can be managed by junior pathologists. This method can help improve the accuracy of pathology diagnosis, save diagnosis time, and ensure diagnostic accuracy.

Although artificial intelligence has played an important role in the medical field, artificial intelligence technology is still not mature enough in the application of digital pathology [4]. Currently, research in the field of pathology primarily focuses on artificial intelligence in areas such as cell segmentation [5], quantitative detection of immunohistochemistry [6], and gene mutation prediction [7]. However, due to the fact that artificial intelligence technology requires robust computer support, and many healthcare professionals lack a foundation in computer science, this may impact the feasibility of intelligent diagnostic models. Our research has adopted a different approach to address this problem. We approached this from a clinical perspective and established predictive models using pathology H&E stained images, avoiding the need for extensive programming expertise and complex program design. Additionally, this model doesn’t require significant computer hardware or powerful server support, making it easier to operate and scale.

## Data and methods

### Image dataset

We collected HE-stained breast tissue slices from the pathology department of Qingdao Central Hospital between 2019 and 2022. We followed the process of dehydration fixation and HE staining, with a slice thickness of 4μm, and included patients with scores above 90 in the study according to the scoring criteria (Table 1) [8]. Digitize these pathology slides through scanning to establish a digital pathology dataset. The use of all slides has been approved through ethical review.

**Table 1:** Routine paraffin-embedded-basic criteria for the quality of HE-stained sections.

number	Premium Standard	full score	Quality Defect Deduction
①	The tissue section is complete, and the number of sections of endoscopic bite and puncture specimens is complete	10	Slightly incomplete organization: minus 1~3 points; incomplete; minus 4~10 points; not reaching the required number of surfaces: minus 5 points
②	Thin slices (3~5μm), uniform thickness	10	Thick slices (overlapping cells), affecting the diagnosis: minus 6~10 points; uneven thickness: minus 3~5 points
③	Slice without knife marks and cracks	10	There are knife marks and fissures, but not affecting the diagnosis: minus 2 points; affecting the diagnosis: minus 5 points
④	Slices are flat, without wrinkles and folds	10	There are wrinkles or folds, but not affecting the diagnosis: minus 2 points; affecting the diagnosis: minus 5 points each
⑤	Slice free of contamination	10	Contaminants: minus 10 points
⑥	No air bubbles (between sections and slides/coverslips and sections and slides), and no glue spills around the coverslips	10	There are bubbles: minus 3 points; glue overflow: minus 3 points
⑦	good transparency	10	Poor transparency: minus 1~3 points; blurred organizational structure: minus 5~7 points
⑧	Clear contrast between nucleus and cytoplasm staining	10	The nucleus is grayish or too blue: minus 5 points; the contrast between red (cytoplasm) and blue (nucleus) is not clear: minus 5 points
⑨	The slices are not loose, and the mounting position is appropriate	10	Loose slices: minus 5 points; improperly mounted slices: minus 5 points
⑩	Neatly sliced, well-labeled, and clearly numbered	10	Untidy slices: minus 3 points; sticky labels: minus 3 points; unclear number: minus 4 points
total		100	

### Image scanning and image analysis

We used the Motic EasyScanner high-definition pathology slide scanner to digitize all the slides. The device is equipped with a fully automated scanning platform, including a main camera, a focusing camera, and a macro camera. The main camera uses a 2/3-inch CCD chip with a resolution of 2448×2048; the focusing camera uses a CCD chip with a resolution of not less than 1360×1024; the macro camera uses a CCD chip with a resolution of 2048×1536. We used Python 3.7 to extract objective data from the images and calculate their R-channel, G-channel, and B-channel values [9] and one-dimensional entropy [10]. One-dimensional entropy can only be calculated from grayscale images. Therefore, we need to preprocess and convert color pathology images to grayscale images to ensure consistency in our calculations.

The average RGB values are a crucial concept widely used in image processing. The average RGB value represents the average of the RGB values of all pixels in an image. RGB refers to the three color channels: red, green, and blue, with each channel ranging from 0 to 255. For an image with N pixels, the RGB average values can be calculated as follows:

avg_R = (R1 + R2 +… + RN) / N

avg_G = (G1 + G2 +… + GN) / N

avg_B = (B1 + B2 +… + BN) / N

**Fig 1:**
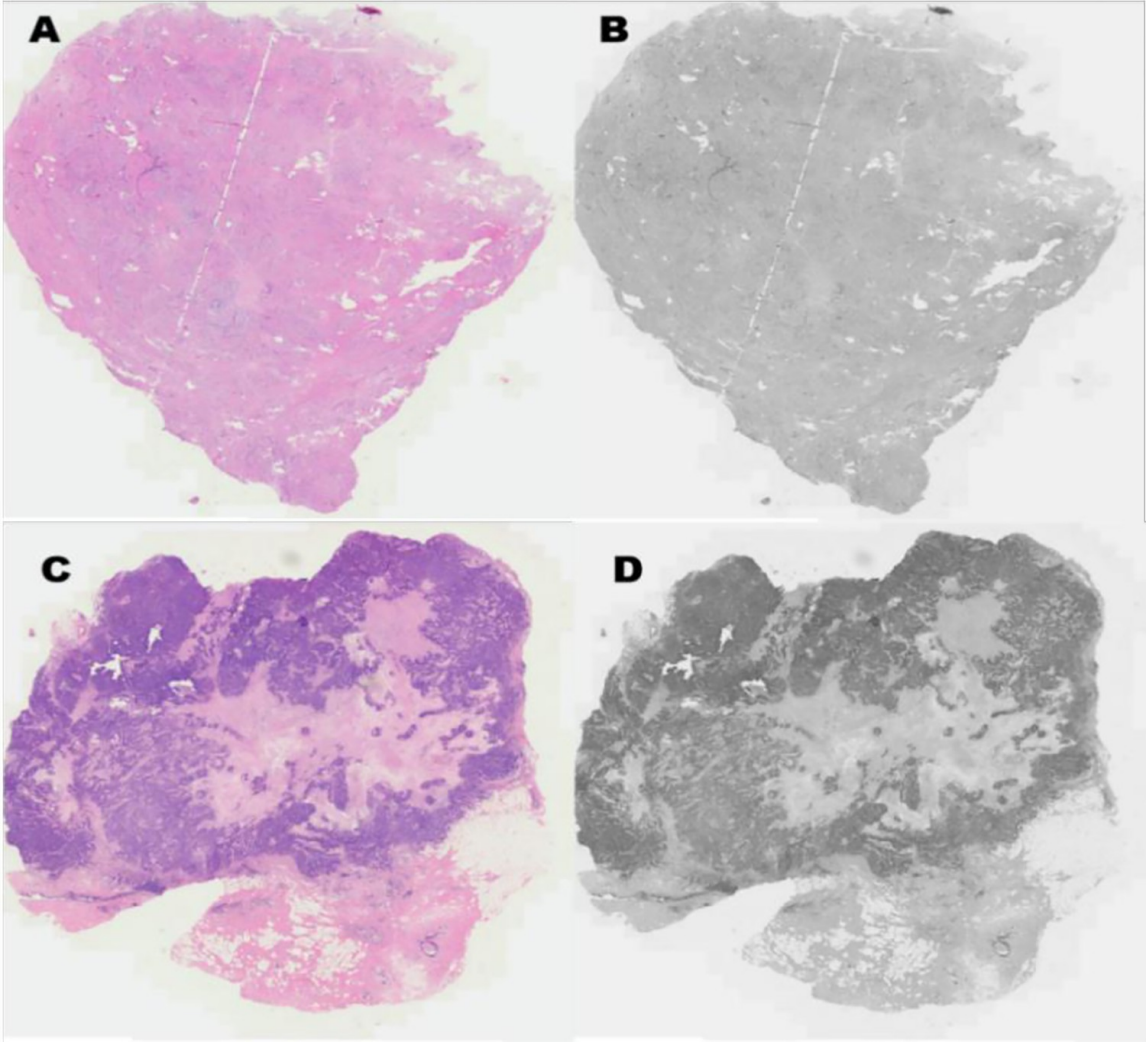
Shows examples of patheological images and grayscale conversion. A and B show images of benign breast tumor tissue and its corresponding grayscale image, while C and D show images of malignant breast tumor tissue and its corresponding grayscale image.

One-dimensional entropy is a statistical measure of feature used to reflect the average amount of information contained in an image (Fig 1). One-dimensional entropy reflects the aggregation characteristics of the grayscale distribution in the image. When calculating one-dimensional entropy, we need to use P_i_ to represent the proportion of pixels with grayscale value i in the image. The one-dimensional entropy calculation formula of gray image is as follows:

$$H=-\sum_{i=0}^{255}{log}_{2}\;P_{i}$$ (1)

### Statistical analysis and nomogram construction

This study used SPSS 25.0 and R 4.2.1 software for data analysis. For normally distributed metric data,`x±s was used to describe and t-test was performed. Variables with statistical significance in the univariate analysis were included in the multiple-factor logistic regression analysis. In the R 4.2.1 software, we used the caret package to randomly divide the data into training and test sets in a 7:3 ratio. Then, we used the rms package to construct a nomogram prediction model, and used the Bootstrap method to calculate the area under the ROC curve (AUC), and Hosmer-Lemeshow test model discrimination and calibration based on 1000 repeated samples. Finally, we used the rmda package for decision curve analysis (DCA) to validate the practicality of the prediction model. All tests were two-sided, with a significance level of α = 0.05, and *P*<0.05 indicated statistical significance.

## Results

### Baseline characteristics

The study collected 2,723 HE stained images from 1474 patients, with an average image size of 0.36M. The breast cancer image dataset contained 2215 images (infiltrating ductal carcinoma), while the breast benign tumor image dataset contained 508 images (adenosis and fibroadenoma). Table 2 shows the basic information of the images in these two datasets. The differences in the R, G, and B channel values, and one-dimensional entropy between the two groups of images were statistically significant (*P*<0.01). In addition, there were no statistically significant differences in the distribution of R (*P* = 0.390), G (*P* = 0.325), B (*P* = 0.317), and one-dimensional entropy (*P* = 0.404) in the training and test sets. These results indicate that there were no significant differences in baseline features between the two groups of images, as shown in Table 3. Tables 4 and 5 are single-factor and multiple-factor regression analyses based on the pathological images in the training group. Based on these results, a nomogram was constructed.

**Table 2:** Compares the image information of benign and malignant breast tumors.

Class	Malignant breast tumor (2214)	Benign breast tumor (509)	*t*	*P-value*
R	219.969±5.137	226.308±6.265	*t* = 24.031	<0.001
G	202.0238±13.060	219.060±17.180	*t* = 24.893	<0.001
B	220.340±8.827	228.996±7.237	*t* = 20.591	<0.001
one-dimensional entropy	5.425±0.573	4.146±0.984	*t* = -38.890	<0.001

**Table 3:** Comparison of image information between training and testing groups.

Class	Total data (2723)	training groups (1907)	testing groups (816)	*t*	*P-value*
R	221.154±5.907	221.218±6.008	221.005±5.664	*t* = 0.859	0.390
G	205.208±15.424	205.399±15.607	204.763±14.987	*t* = 0.985	0.325
B	221.958±9.193	222.054±9.202	221.733±9.174	*t* = 0.834	0.317
one-dimensional entropy	5.186±0.834	5.172±0.853	5.219±0.789	*t* = -1.346	0.404

**Table 4:** Presents the results of the single-factor regression analysis based on the modeling group data.

Class	Malignant breast tumor (1541)	Benign breast tumor (366)	*t*	*P-value*
R	219.908±5.226	226.730±5.965	*t* = -21.825	<0.001
G	201.866±13.183	220.272±16.253	*t* = -22.896	<0.001
B	220.278±8.825	229.530±6.650	*t* = -18.825	<0.001
one-dimensional entropy	5.430±0.579	4.084±0.958	*t* = 34.653	<0.001

**Table 5:** Presents the results of the multiple-factor regression analysis based on the modeling group data.

Independent variable	*β*	*SE*	*Waldχ* ^2^	*P*	*OR*	*OR* 95%CI
R	0.226	0.032	50.028	<0.001	1.254	1.178–1.335
B	-0.073	0.018	16.911	<0.001	0.930	0.898–0.963
one-dimensional entropy	2.998	0.201	222.808	<0.001	20.047	13.523–29.718
Constant	-47.265	6.657	50.406	<0.001	-	-

### Model construction and verification

**Fig 2:**
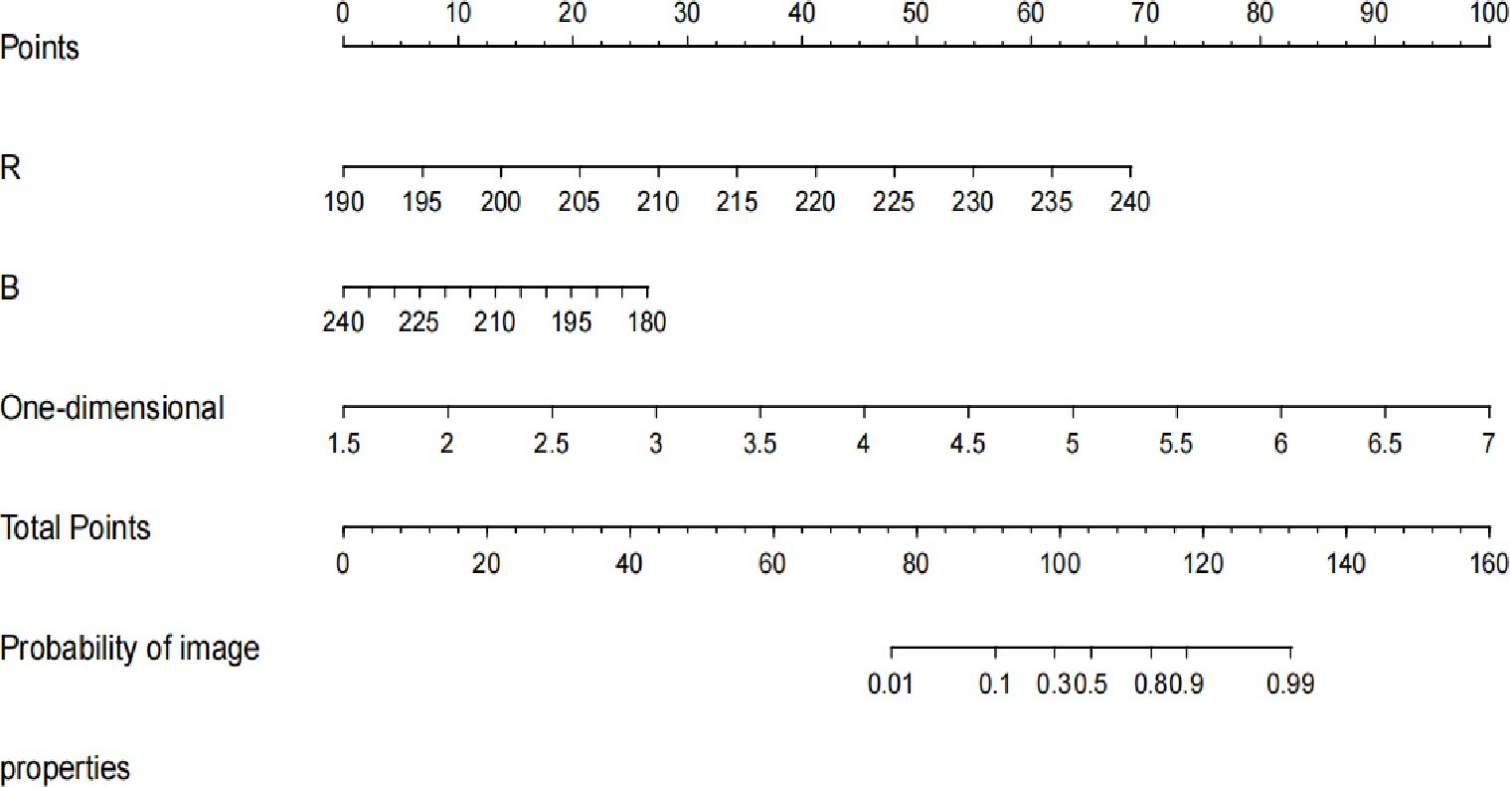
Nomogram of pathological type prediction. Nomogram including the variables of R, B, and one-dimensional entropy.

**Fig 3:**
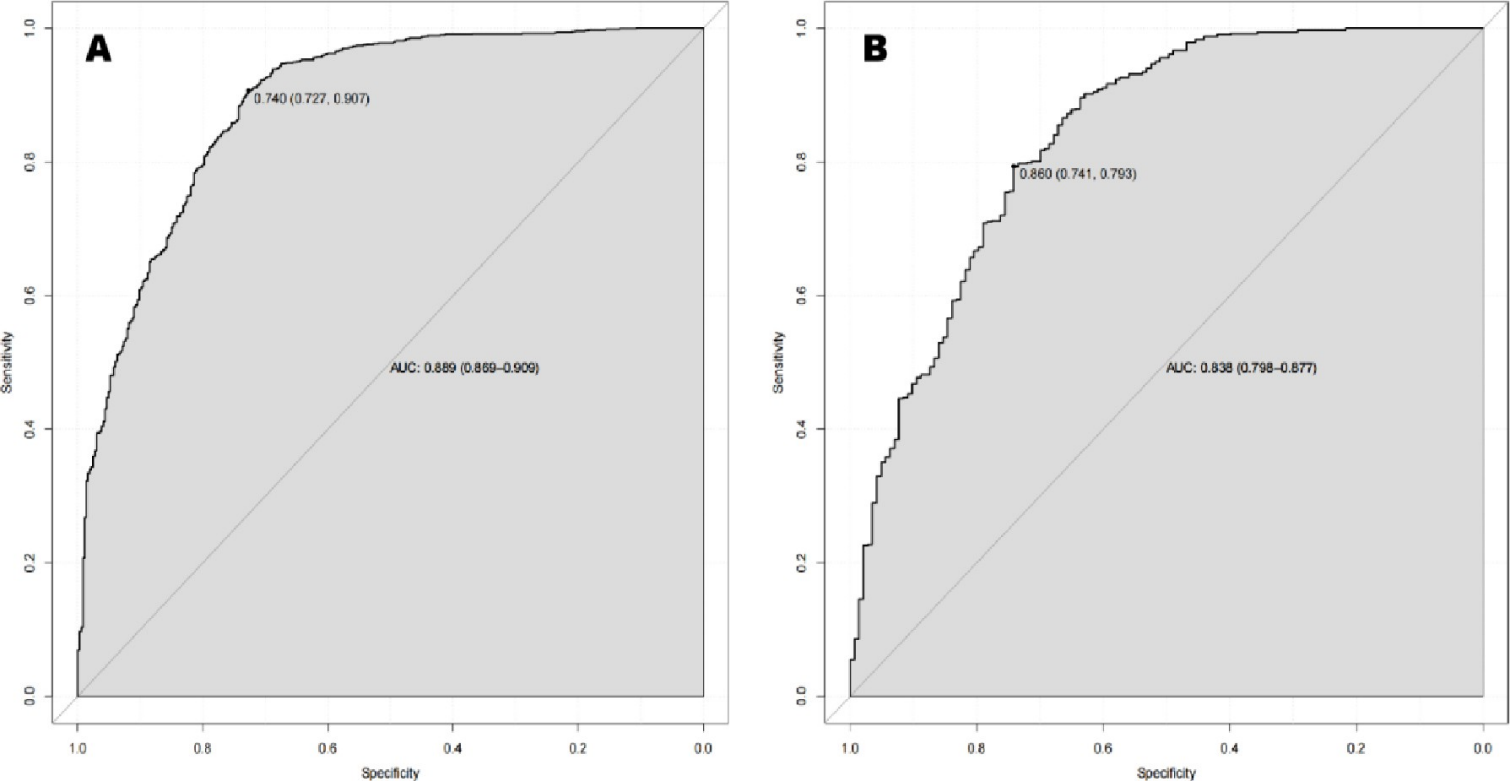
Nomogram of AUC area. A: The AUC result of the nomogram was 0.889 (95% CI: 0.869, 0.909) in the training set. B: The AUC result of the nomogram was 0.838 (95% CI: 0.798, 0.877) in the validation set.

**Fig 4:**
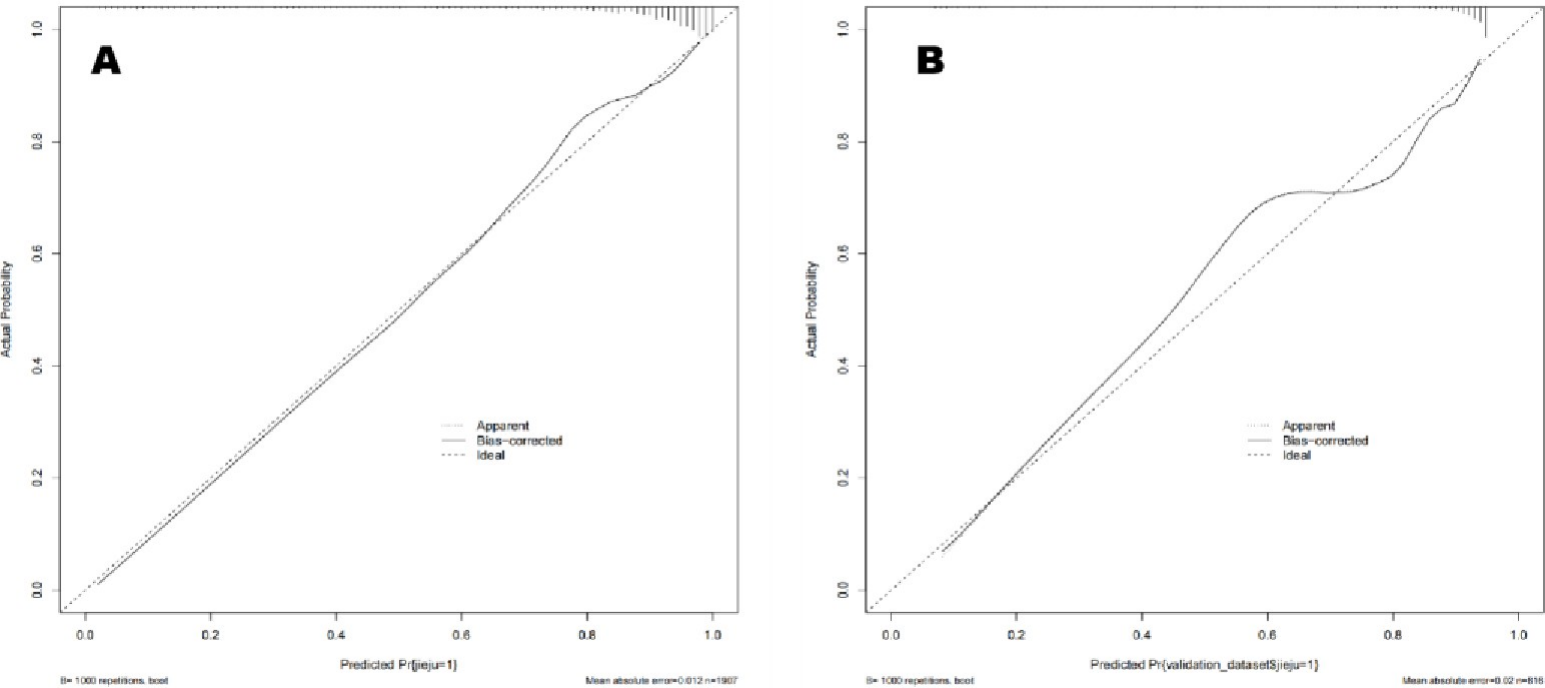
Nomogram of the calibration curve. A represents the training group, and B represents the validation group. The calibration curve shows that the nomogram model has good discrimination and accuracy in distinguishing between benign and malignant pathological tissues in both the training and validation sets.

**Fig 5:**
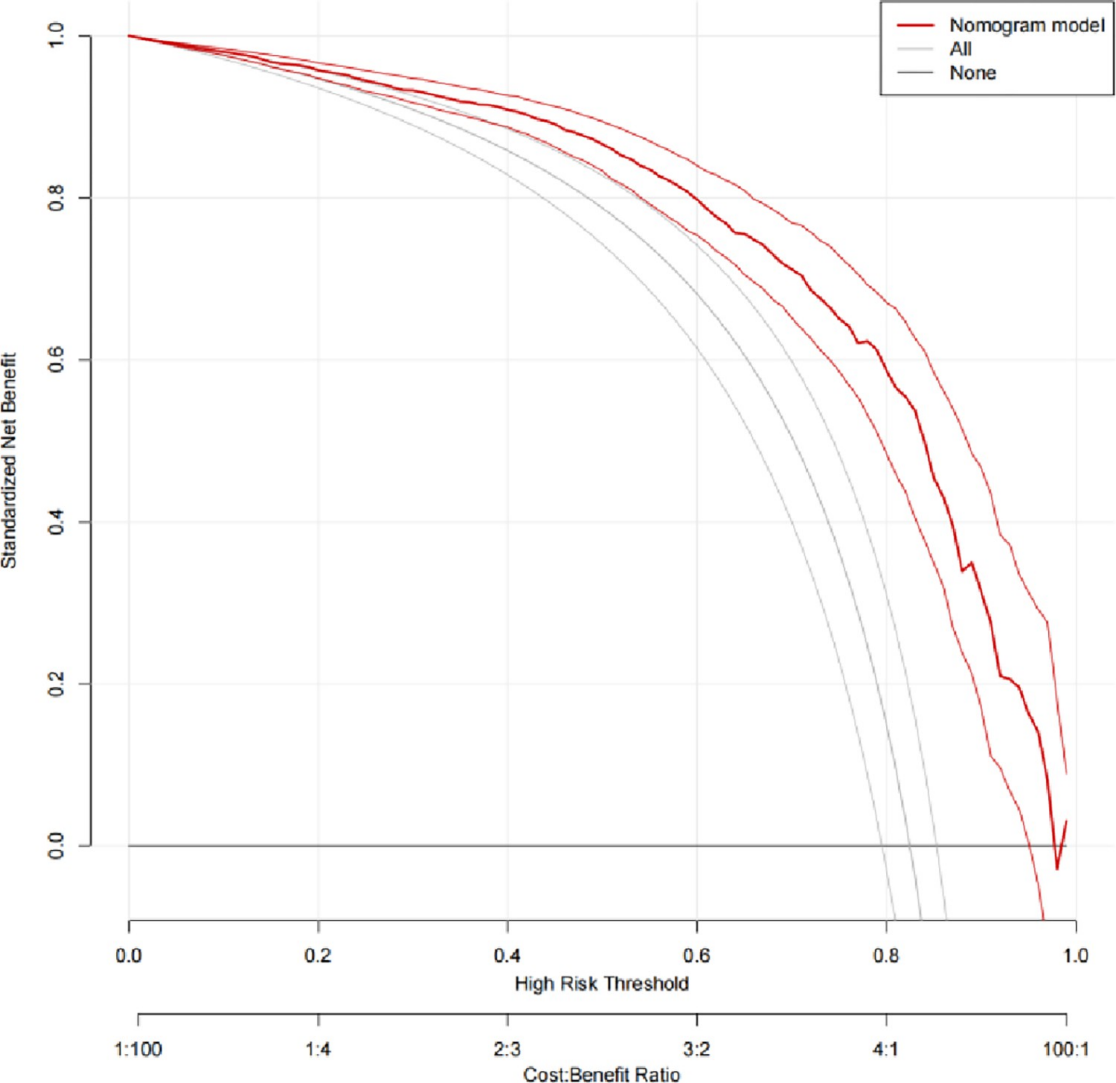
Decision curve analysis. Construct decision curves for prediction models in the validation set. In the probability threshold range of more than 40%, the net benefit value of the prediction model curve of the nomogram was higher than the extreme line, which validated its practicality.

The performance of the nomogram in distinguishing between malignant and benign breast lesions, with a Hosmer-Lemeshow test result of *χ*^*2*^ = 10.150 (*P* = 0.255) (Fig 2). The AUC result of the nomogram was 0.889 (95% CI: 0.869, 0.909) in the training set and 0.838 (95% CI: 0.798, 0.877) in the validation set (Fig 3). The calibration curves of the nomogram in the training and validation sets are shown in Fig 4. In predicting breast malignant tumors, the nomogram showed good consistency with histopathological diagnosis. We plotted the decision curve of the validation set in Fig 5, with the middle line representing the prediction curve and the two lines before and after representing the 95% confidence interval. In the probability threshold range of more than 40%, the net benefit value of the prediction model curve of the nomogram was higher than the extreme line, which validated its practicality.

## Discussion

Pathologists’ diagnosis of tissue slides is the cornerstone of disease diagnosis [11]. Histopathological slides stained with Hematoxylin and Eosin (H&E) have been used for over a century and still remain the standard staining method for routine tissue pathology diagnosis [12]. Digital pathology enables quantitative analysis of images through the digitization of microscopic images. While other imaging disciplines have been computer-based for many years [13–15], the digitization of pathology is only just beginning. With the introduction of whole slide imaging (WSI), the field of pathology has been able to generate large-scale digital datasets. Deep learning has ushered in a new era in the field of general object classification and detection [16, 17]. The classification of tumor tissue histopathology images has always been an important area of research. Babak Ehteshami Bejnordi et al. [18]. achieved an accuracy of 0.924 in a binary classification task using convolutional neural networks on a pathology image dataset of size 224×224. Muhammad Sadiq Amin et al. [19] achieved an accuracy of 0.92 in a binary classification task on the BreakHis dataset using DenseNet. High-resolution histopathology images have made traditional machine learning algorithms and deep neural network models for viewing whole slide images (WSI) extremely complex [20]. Furthermore, the limited number of samples for cancer tissue histopathology image classification and the large image sizes make training deep learning models challenging. Additionally, compressing the entire tumor image array to fit the input size of the model leads to a loss of rich detailed feature information. In this study, the binary classification AUC was 0.838 (95% CI: 0.798, 0.877). Although this accuracy is not higher than previous research, its operability is very strong and it is easy to create and apply.

The application of digital pathology in traditional pathology is greatly limited by factors such as computer hardware, processing time, image analysis methods, and data storage. We utilize quantitative image analysis (QIA) mathematical operations to analyze pathological images based on color, texture, and other information. This predictive model is not only suitable for limited medical electronic devices but also contributes to improving the accuracy of pathological diagnosis. Compared to previous deep learning models [21–23], our research focuses primarily on the benign-malignant classification of whole slide pathology images. We have developed and externally validated a new practical model consisting of three variables, including the values of the R and B channels and one-dimensional entropy, all calculated through program code. The model has been externally validated on a test set and demonstrates good identification and predictive performance. Future research may improve predictive factors by selecting additional feature variables such as tumor markers and inflammatory factors.

Some pathological laboratories have integrated WSI scanners into their routine workflow to enable digital diagnostic workflows, and the consistency between digital image diagnosis and traditional slide diagnosis has been shown to be superior. However, finding a solution that provides sufficient storage capacity and reasonable archiving costs remains a challenge. Breast pathology has had years of experience with auxiliary diagnostic software that uses image processing techniques to extract image features, and many scholars currently adopt deep learning-based methods [24]. However, tissue pathology image analysis methods can only analyze small regions of interest (ROIs), rather than large-scale whole slide images (WSIs) [25]. According to the new image analysis model development strategy, pathologists may need to manually annotate digital slide images, which is both time-consuming and subjective, and can lead to observer differences even among senior pathologists [26]. Studies have shown that in certain tasks, artificial intelligence can perform as well as human experts, but there are still limitations and many challenges for artificial intelligence. Clinical applications are expected to develop slowly because (i) the lack of interpretable models in the medical community may hinder doctors’ trust [27]. If doctors cannot understand the reasons why algorithms make decisions, they may ignore the decisions of intelligent diagnostic models, thereby limiting their practicality. (ii) The cost of setting up digital slide scanners, image storage, image analysis software, and IT support systems is high. (iii) The diagnostic results of artificial intelligence must be continuously and prospectively verified over a certain period of time.

In the field of pathological image processing, deep learning models have become a widely used technology. However, existing deep models mostly only process small ROI regions and lack a global perspective, while the generalization problem is also a challenge [28]. Pathological images from different centers can exhibit variations that lead to model instability. While scholars have suggested image normalization as a potential solution [29], the results are often insufficient for the needs of pathology. To address these issues, we propose a new approach of building a custom model to tackle the diversity of images in different pathological centers. This method does not require expensive computing power and storage devices, nor does it require additional expenses for pathological centers, as the construction of the model is very easy and can even be done by pathologists. Although deep learning models have generalization problems, we believe that with advances in computer technology, standardization of staining processes, and lossless image transmission technologies, artificial intelligence technology will have a leap forward. Currently, we can still use assisted diagnostic software to help pathologists improve diagnostic accuracy and reduce their workload [30, 31]. Although manual annotation is still a tedious and subjective task, we can still use artificial intelligence technology to alleviate the workload of doctors and improve their diagnostic efficiency. In conclusion, we believe that through continuous experimentation and innovation, the digitization of the field of pathology will become more mature and complete.

## Conclusion

The proposed prediction model in this study demonstrates ideal predictive results and can provide effective suggestions in the diagnostic process of pathologists, improving diagnostic efficiency and accuracy. However, we also acknowledge that there are some limitations to this prediction model. Since the images used in this study were whole slide images with poor resolution, the fine structural features of the images were lost while improving efficiency. Benign diseases tend to be treated conservatively, so fewer benign pathological tissue slices are obtained. The ratio of malignant tumors to benign tissues used to construct the model is 4:1, which may increase the instability of the model. We will continuously collect images of benign tumor tissue to improve our research. Therefore, our next research direction is to combine this prediction model with deep learning models to improve the diagnostic efficiency and accuracy of deep learning models.

## Supporting information

S1 File Code for grayscale image conversion. (DOCX)

S2 File Code for computing one-dimensional entropy of an image. (DOCX)

S3 File Code for computing R, G, B values of a HE-stained image. (DOCX)
