# Supplementary material for: A prediction model based on digital breast pathology image information
Source: PLoS One. 2024 May 17;19(5):e0294923. doi: 10.1371/journal.pone.0294923 (PMC11101065; doi:10.1371/journal.pone.0294923)
Supplement: S1 File — (DOCX) [file pone.0294923.s001.docx]

from PIL import Image
import os
path = 'C:/Users/hp/Desktop/123'
file_list = os.listdir(path)
for image in file_list:
 I = Image.open(path + "/" + image)
 gray = I.convert('L')
 gray.save(path + "/" + image)
 #print(file)
