# Supplementary material for: A prediction model based on digital breast pathology image information
Source: PLoS One. 2024 May 17;19(5):e0294923. doi: 10.1371/journal.pone.0294923 (PMC11101065; doi:10.1371/journal.pone.0294923)
Supplement: S2 File — (DOCX) [file pone.0294923.s002.docx]

import math
import cv2
import numpy as np
import os
from PIL import Image


# image = cv2.imread('C:/Users/hp/Desktop/123/2113636g2.png', 0)
# img = np.array(image)
# ===========================
img_path = 'C:/Users/hp/Desktop/jrRGB/'
file_list = os.listdir(img_path)
# ===========================
for file_name in file_list:
 img = Image.open(img_path + file_name)
 img = np.array(img)
 res = 0
 tmp = [0] * 256
 val = 0
 k = 0
 for i in range(len(img)):
 for j in range(len(img[i])):
 val = img[i][j]
 tmp[val] = float(tmp[val] + 1)
 k = float(k + 1)
 for i in range(len(tmp)):
 tmp[i] = float(tmp[i] / k)
 for i in range(len(tmp)):
 if tmp[i] == 0:
 res = res
 else:
 res = float(res - tmp[i] * (math.log(tmp[i]) / math.log(2.0)))
 print(file_name, res)
