# Supplementary material for: A prediction model based on digital breast pathology image information
Source: PLoS One. 2024 May 17;19(5):e0294923. doi: 10.1371/journal.pone.0294923 (PMC11101065; doi:10.1371/journal.pone.0294923)
Supplement: S3 File — (DOCX) [file pone.0294923.s003.docx]

import os
import numpy as np
import cv2

ims_path = 'C:/Users/hp/Desktop/nyt/'
ims_list = os.listdir(ims_path)
R_means = []
G_means = []
B_means = []
for im_list in ims_list:
 im = cv2.imread(ims_path + im_list)
 # extrect value of diffient channel
 im_R = im[:, :, 0]
 im_G = im[:, :, 1]
 im_B = im[:, :, 2]
 # count mean for every channel
 im_R_mean = np.mean(im_R)
 im_G_mean = np.mean(im_G)
 im_B_mean = np.mean(im_B)
 # save single mean value to a set of means
 R_means.append(im_R_mean)
 G_means.append(im_G_mean)
 B_means.append(im_B_mean)
 print('图片：{} 的 RGB平均值为 [{}，{}，{}]'.format(im_list, im_R_mean, im_G_mean, im_B_mean))
# three sets into a large set
a = [R_means, G_means, B_means]
mean = [0, 0, 0]
# count the sum of different channel means
mean[0] = np.mean(a[0])
mean[1] = np.mean(a[1])
mean[2] = np.mean(a[2])
print('数据集的BGR平均值为\n[{}，{}，{}]'.format(mean[0], mean[1], mean[2]))
